# Optimization Research on Sensor Network Layout for Microseismic Monitoring Based on Location Error Analysis

**DOI:** 10.3390/s26144533

**Published:** 2026-07-17

**Authors:** Xiaofeng Huang, Shenglan Li, Longjun Dong, Longbing Yang

**Affiliations:** School of Resources and Safety Engineering, Central South University, Changsha 410083, China; 195502037@csu.edu.cn (X.H.); 255503031@csu.edu.cn (S.L.); 225507003@csu.edu.cn (L.Y.)

**Keywords:** microseismic monitoring, sensor network optimization, localization error, geometric configuration, Geiger algorithm, deep mining

## Abstract

This study develops a numerical framework for optimizing microseismic sensor network layouts in the Woxi Mine. Three candidate deployment schemes were evaluated by combining synthetic arrival-time perturbations with the classical Geiger localization algorithm under different uncertainty levels. The results show that localization performance is governed primarily by sensor layout geometry, which controls both the magnitude and the spatial distribution of localization errors. Event-wise robustness analysis indicates that the layout with the lowest overall localization error is not necessarily the one with the strongest spatial enclosure. These findings provide a geometry-based reference for the preliminary design and subsequent refinement of a mine-scale microseismic monitoring system in deep underground engineering.

## 1. Introduction

Deep underground engineering and mineral resource extraction frequently encounter highly complex environments characterized by high stress, elevated temperatures, and significant seepage. Under the coupled effects of multiple factors, including mining disturbances and blasting, the surrounding rock mass becomes susceptible to progressive damage and potential dynamic disasters. Acoustic emission (AE) and microseismic (MS) monitoring technologies, which characterize the rock mass fracturing process through spatiotemporal and energy evolution, have emerged as essential tools for disaster identification and early risk warning [1,2,3,4]. Extensive studies have investigated rock mass fracturing mechanisms, crack propagation, and energy release patterns under various stress paths to date [5,6,7,8,9]. International applications have also demonstrated the engineering value of microseismic monitoring in underground mining and rock mass stability assessment, with representative case studies reported in Canadian underground mines, the MMG Century Mine in Australia, and deep South African gold mines [10,11,12]. Under deep mining conditions, micro-fracture events occur frequently, and wave propagation paths are inherently complex. Consequently, MS signals are particularly vulnerable to strong background noise and medium heterogeneity, posing significant challenges to the deployment of reliable monitoring systems.

The reliability of MS monitoring and location is controlled by key links: “sensor deployment—arrival time picking—velocity model—location inversion.” Among these, the spatial geometric configuration of the sensor network determines the coverage and intersection angle of ray paths, directly limiting the upper bound of location accuracy and the spatial distribution of errors. Studies indicate that sensor quantity, geometric configuration, and source-sensor distance systematically affect location errors [13], and optimizing the spatial layout is essential to reduce the amplification of these errors [14,15,16]. Simply increasing the number of sensors does not necessarily yield a proportional improvement in location accuracy; in areas with restricted deployment, poor geometric configurations often lead to exceptionally high location errors. Restricted by construction conditions, geological structures, and spatial scales in underground engineering, sensor layout rarely achieves an ideal geometric configuration [17]. Therefore, it is urgent to quantitatively analyze the control mechanisms of deployment schemes on the distribution of location errors under real engineering constraints.

In addition to array geometry, arrival time picking error is another core variable affecting location reliability. High background noise in deep mines significantly increases the uncertainty of first-arrival picking [18,19]. Existing studies have developed advanced signal recognition and picking algorithms, such as instantaneous phase difference [6], wavelet analysis [20], and deep learning [21,22,23], along with improved Short-Term Average/Long-Term Average (STA/LTA) and Akaike Information Criterion (AIC) combinations [24], revealing the non-stationary characteristics of picking errors that dynamically change with lithology, stress, and noise. Regarding location inversion, the classical Geiger’s method based on minimizing arrival time residuals [25] and the double-difference location algorithm [26] have laid the theoretical foundation [27]. Location without a pre-set wave velocity [28,29] and cutting-edge AI intelligent location methods [23,30], as well as metaheuristic-optimized grid search and 3D travel time field models [31,32,33], have further expanded the inversion boundaries in complex scenarios. Although intelligent algorithms perform exceptionally well on specific datasets, their engineering generalization capabilities remain limited by the distribution shift in training data. During the design phase of a monitoring system, it is essential to return to the physical root of “array geometry + error propagation” to explore what spatial distribution can maintain the strongest location robustness against variable arrival time errors under given installation constraints.

To address these engineering challenges, this study quantitatively evaluates the localization performance of different microseismic sensor network layouts under multiple arrival-time uncertainty levels. Using the deep mining section of the Woxi Mine as the engineering background, three three-dimensional sensor network schemes were designed by considering site-specific constraints, including monitoring area geometry, tunnel distribution, and elevation differences. A numerical model was then established to introduce graded random arrival-time errors (0–2%, 2–5%, 5–8%, and 8–10%), and the classical Geiger iterative algorithm was adopted as a unified inversion method. On this basis, the study examines how array geometry influences the magnitude of localization error, the variation in the spatial error pattern with increasing arrival-time uncertainty, and layout robustness across different mining levels. The results provide a quantitative reference for the preliminary optimization of candidate monitoring nodes in the Woxi Mine. In addition to the spatial error maps, the present study further evaluates layout performance using event-wise robustness statistics and geometry-based metrics. Although sensor-network optimization in mines has been studied previously [15,17], existing work largely evaluates a chosen configuration in terms of overall location accuracy. The present study differs in that it treats arrival-time uncertainty as a controlled variable and quantifies how the spatial error pattern and event-wise robustness of competing layouts respond to increasing timing error across multiple source depths. The main contributions are: (i) a graded-uncertainty, error-propagation framework for comparing candidate layouts; (ii) the finding that the lowest-error layout is not necessarily the one with the strongest spatial enclosure, with the controlling factor shifting from local geometric matching to global enclosure as uncertainty increases; and (iii) a robustness-based, geometry-linked evaluation that yields an explicit, conditional deployment recommendation for the deep section of the Woxi mine.

## 2. Materials and Methods

### 2.1. Brief Description of Geological Conditions

The Woxi Gold-Antimony-Tungsten Mine is located in Yuanling County, Hunan Province. The regional topography is characterized by the Xuefengshan arcuate uplift belt, where the Proterozoic Lengjiaxi (Ptl) and Banxi (Ptb) Groups of epimetamorphic rocks form the basement. The deposit is a low-to-moderate temperature hydrothermal quartz vein type, primarily consisting of several gently dipping parallel ore veins. Individual orebodies extend from dozens to hundreds of meters along the strike, with an average dip angle of approximately 26°. The surrounding rock mainly comprises purple-red slate and shale, with a Protodyakonov hardness coefficient (f) ranging from 6 to 8.

**Figure 1:**
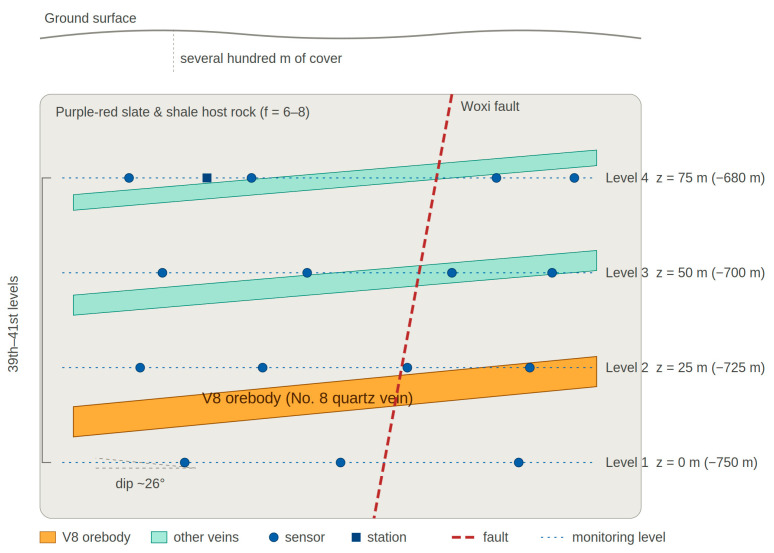
Schematic of the geological and monitoring background of the deep section of the Woxi mine (vertical cross-section, not to scale). The gently dipping parallel quartz veins (including the V8 orebody) are hosted in purple-red slate and shale and are cut by the Woxi fault. Four monitoring levels (z = 0, 25, 50, and 75 m, corresponding to the actual mine elevations of approximately −750, −725, −700, and −680 m) span the 39th–41st mining levels, where sensors (circles) and acquisition stations (squares) are deployed along the accessible tunnels.

The mining area is structurally complex, dominated by the major Woxi fault, transverse faults, and interlaced fractured zones. These structures, accompanied by dense joint networks and veinlet zones, result in highly heterogeneous geological media with volatile mechanical properties. In the deep mining sections (the 39th to 41st levels, corresponding to approximately −750 m to −680 m in elevation, a vertical interval of about 70 m), the geomechanical environment of the V8 orebody (the No. 8 gently dipping quartz-vein orebody, which is the principal orebody mined in the deep section) is particularly challenging. Frequent stress redistribution induced by excavation and blasting leads to high stress concentration, triggering rock mass failures such as sliding, shearing, or fracturing along structural planes. Under conditions of significant burial depth and intense structural fragmentation, frequent ground pressure activities—including roof falls, rib spalling, and potential rockbursts—pose severe threats to the safety and efficiency of deep mining operations. The geological and monitoring background of the deep section is shown in Figure 1.

### 2.2. Sensor Network Layout Design

In practical underground engineering, sensor deployment needs to satisfy both monitoring requirements and engineering feasibility [10,11]. The layout design in this study was developed with consideration of the spatial extent and geometric morphology of the monitoring area, the distribution of tunnels, stopes, and geological structures, the accessibility of sensor installation and maintenance, and the vertical separation required for three-dimensional monitoring. Within these constraints, the sensor network was optimized for the target monitoring volume of the Woxi mine.

To strengthen the spatial enclosure of the monitoring volume, the monitoring system was organized into multiple four-sensor groups. In each group, four sensors were connected to one acquisition station, forming a local enclosing unit in the three-dimensional network. In the layout figures, circular symbols denote sensors, whereas rectangular symbols denote acquisition stations. The acquisition stations are labeled A, B, C, D, E, F, and G, and the sensors connected to each station are identified using corresponding group labels such as A1, A2, A3, and A4. Accordingly, the number of sensors deployed at each mining level was set as a multiple of four. Group labels in the layout figures are used to identify different sensor groups and their associated acquisition stations; colors are used only as visual aids.

A local three-dimensional Cartesian coordinate system was established for the target monitoring volume of the Woxi mine. A local vertical coordinate z was defined with its origin (z = 0 m) placed at the lowest monitored level, and the four monitoring levels were thus located at z = 0, 25, 50, and 75 m, which correspond to the actual mine elevations of approximately −750, −725, −700, and −680 m, respectively. Hence, z = 0 m (the elevation datum), the first monitoring level, and the −750 m mine elevation all denote the same horizon. All sensor coordinates, source coordinates, and localization calculations reported in this study are expressed in this local reference system.

Three candidate microseismic monitoring layouts, denoted as Scheme I, Scheme II, and Scheme III, were designed for comparative evaluation [15,17]. To ensure a fair comparison, all three schemes contain the same total number of sensors and the same number of nodes at each monitoring level, thereby forming comparable three-dimensional monitoring networks under different geometric configurations.

**Figure 2:**
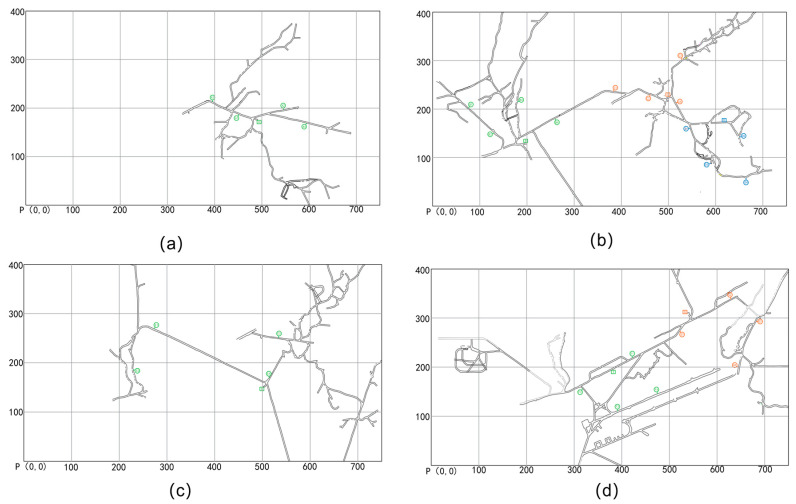
Sensor network layout for Scheme I: (**a**) 39th level (z = 75 m, −680 m); (**b**) 40th level (z = 50 m, −700 m); (**c**) 41st level (z = 25 m, −725 m); (**d**) 42nd level (z = 0 m, −750 m). Circular symbols denote sensors, rectangular symbols denote acquisition stations, and group labels identify the corresponding four-sensor groups.

Scheme I represents the baseline layout. The sensor groups are mainly distributed along both sides of the main tunnel. A total of 8, 4, 12, and 4 sensors are arranged from the first to the fourth monitoring levels, respectively, as shown in Figure 2. This layout is convenient for installation and maintenance, but its spatial enclosure of the monitoring volume is relatively limited.

**Figure 3:**
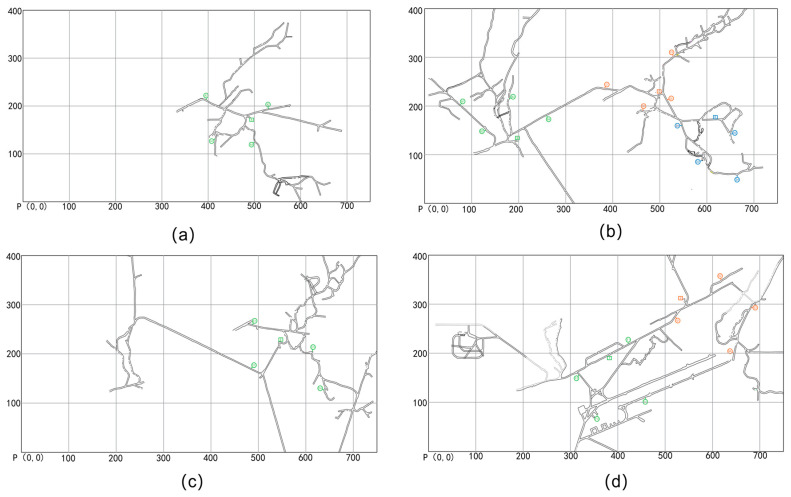
Sensor network layout for Scheme II: (**a**) 39th level (z = 75 m, −680 m); (**b**) 40th level (z = 50 m, −700 m); (**c**) 41st level (z = 25 m, −725 m); (**d**) 42nd level (z = 0 m, −750 m). Circular symbols denote sensors, rectangular symbols denote acquisition stations, and group labels identify the corresponding four-sensor groups.

Compared with Scheme I, Scheme II redistributes several four-sensor groups to improve the geometric balance of the network, especially at the lower and intermediate monitoring levels. In this scheme, some groups originally concentrated along both sides of the main tunnel were moved toward more outward and dispersed positions so that the array forms a more continuous three-dimensional enclosure of the monitoring volume. These adjustments reduce the local concentration of sensors in Scheme I and improve the effective coverage of the central and boundary regions of the monitoring area, as shown in Figure 3.

**Figure 4:**
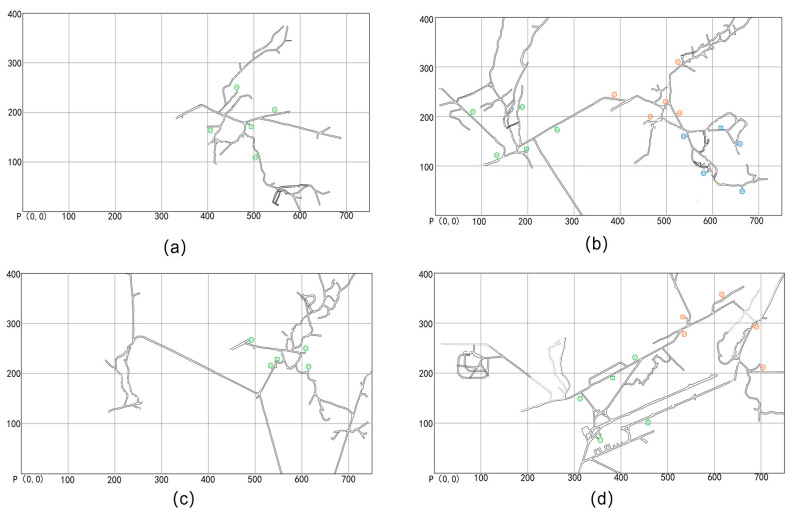
Sensor network layout for Scheme III: (**a**) 39th level (z = 75 m, −680 m); (**b**) 40th level (z = 50 m, −700 m); (**c**) 41st level (z = 25 m, −725 m); (**d**) 42nd level (z = 0 m, −750 m). Circular symbols denote sensors, rectangular symbols denote acquisition stations, and group labels identify the corresponding four-sensor groups.

Scheme III was developed by further adjusting several four-sensor groups at the lowest and highest monitoring levels, particularly at z = 0 m and z = 75 m. Compared with Scheme II, these groups were redistributed toward more outward positions near the lower and upper boundaries of the monitoring volume so as to strengthen the three-dimensional enclosure of the network. This arrangement improves boundary coverage, extends the effective monitoring range in the vertical direction, and enhances the overall spatial continuity of the layout, as shown in Figure 4.

The differences in spatial configurations among the three schemes provide the basis for the subsequent comparative analysis of localization errors.

### 2.3. Numerical Model and Source Localization Method

The numerical evaluation procedure consisted of four main steps: synthetic source generation, theoretical arrival-time calculation, arrival-time perturbation, and Geiger-based source localization.

To quantitatively evaluate the performance of the three proposed sensor network layouts, localization accuracy was used as the primary evaluation index. A three-dimensional numerical model was established for the target monitoring volume, incorporating the three candidate sensor network schemes described above. Within this model, three layers of synthetic microseismic sources were randomly generated, with 50 sources in each layer, resulting in a total of 150 simulated events for each layout scheme. The three source layers were arranged as horizontal planes at the lower, middle, and upper parts of the monitoring volume (approximately z = 12.5, 37.5, and 62.5 m, interleaved between the four sensor levels), and within each layer, the 50 sources were randomly distributed over the horizontal (x–y) extent of the monitoring area.

The rock mass was simplified as a homogeneous and isotropic medium with a constant wave velocity of 4000 m/s. Under this assumption, the theoretical arrival time from source i to sensor j is given by:

(1) $$t_{ij}=\frac{sqrt\left({\left(x_{i}-x_{j}\right)}^{2}+{\left(y_{i}-y_{j}\right)}^{2}+{\left(z_{i}-z_{j}\right)}^{2}\right)}{v}$$

where $\left(x_{i},y_{i},z_{i}\right)$ and $\left(x_{j},y_{j},z_{j}\right)$ are the coordinates of the source and sensor, respectively, and v is the wave velocity.

To represent arrival-time uncertainty in the numerical model, a symmetric random percentage perturbation was applied to each theoretical arrival time. For a given error interval [a, b], the perturbed arrival time was defined as

(2) $$t_{ij}^{\prime }=t_{\left(ij\right)\left(1+epsilon_{ij}\right)}$$

where $\varepsilon_{ij}$ is expressed as

$$epsilon_{ij}=s_{ij}delta_{ij}$$

Here, $\delta_{ij}$ follows a continuous uniform distribution on [a, b], and $s_{ij}$ takes −1 or +1 with equal probability. Therefore, the perturbation is multiplicative, the perturbation magnitude is uniformly sampled from the prescribed percentage interval, and the perturbation direction is randomly assigned as positive or negative. Four arrival-time uncertainty levels were considered in this study: 0–2%, 2–5%, 5–8%, and 8–10%.

Source localization was carried out using a Python 3.14.0-based implementation of the classical Geiger iterative algorithm. The classical Geiger algorithm was adopted as a unified, deterministic inversion method because the objective of this study is to isolate the influence of array geometry and arrival-time error propagation on localization accuracy; as a transparent, widely validated absolute-location method that requires no training data or event clustering, it ensures that performance differences among the layouts reflect geometry and timing error rather than algorithm-specific behavior. Relative methods such as the double-difference algorithm and data-driven deep-learning locators are valuable for field application and will be considered in subsequent validation work. For each simulated event, the source position and origin time were obtained by minimizing the residuals between the perturbed arrival times and the theoretical arrival times calculated from the trial source coordinates. Let the source location be denoted by $S\left(x_{0},\;y_{0},z_{0}\right)$ and the origin time by $t_{0}$. The objective function is expressed as

(3) $$Phi\left(t_{0},x_{0},y_{0},z_{0}\right)=sum_{i=1}^{n}r_{i}^{2}$$

where the residual $r_{i}$ is defined as

$$r_{i}=t_{0}+T_{i\left(x_{0},y_{0},z_{0}\right)}-t_{i}^{\prime }$$

In this expression, $r_{i}$ is the residual between the calculated arrival time and the perturbed arrival time at sensor i, $T_{i}\left(x_{0},\;y_{0},\;z_{0}\right)$ is the calculated travel time from the trial source position to sensor i, and n is the number of sensors used in the inversion; in this study, all available sensors in each layout scheme were included for every synthetic event. A smaller value of Φ indicates a better fit between the calculated and perturbed arrival times.

The initial source position was set at the geometric center of the monitoring volume, and the initial origin time was estimated from the earliest corrected arrival time as

(4) $$t_{0}^{0}=\underset{i}{\min}\left[t_{i}^{\prime }-T_{i\left(x_{0}^{0},y_{0}^{0},z_{0}^{0}\right)}\right]$$

The Geiger iteration was terminated when the Euclidean norm of the position update became smaller than 10^−3^ m or when the change in the objective function became smaller than 10^−8^; otherwise, the inversion stopped after 50 iterations. No explicit outlier rejection was applied because the analysis was based on controlled synthetic arrival-time data and focused on the influence of array geometry and error propagation. The localization error was defined as the three-dimensional Euclidean distance between the inverted source position and the theoretical source position. This value was used to characterize localization accuracy and to compare the robustness of different sensor network layouts under different arrival-time error levels.

## 3. Results

### 3.1. Localization Error Distribution Characteristics

Under the theoretical (unperturbed) arrival-time condition, all three schemes recovered the true source coordinates to within the numerical tolerance of the inversion, with localization errors on the order of 10^−12^ m (i.e., effectively zero). These residual values represent numerical round-off rather than a physical error; this case is therefore used only as the reference baseline for the subsequent analysis.

**Figure 5:**
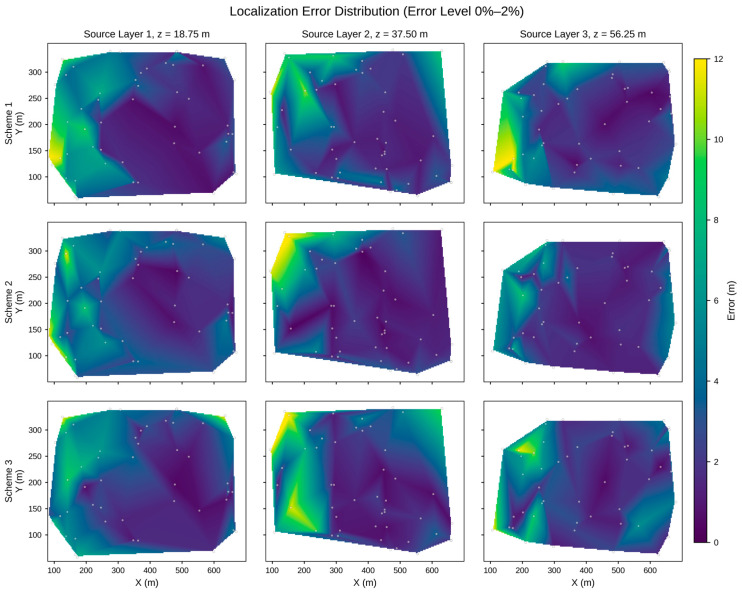
Localization error distribution of different layout schemes under arrival time errors of 0–2%.

When the arrival-time error ranged from 0% to 2%, the localization error distributions of the three monitoring schemes are shown in Figure 5. For the first source layer, Scheme III achieved the lowest mean localization error (3.9 m), with about 85% of the monitoring area remaining below 6.2 m and the larger errors confined mainly to the upper-left and lower-left boundaries. Schemes I and II showed higher mean errors (4.2 m and 4.0 m, respectively), with more pronounced high-error regions along the lower-left and middle-left boundaries.

For the second source layer, Scheme II again provided the most favorable performance, with a mean error of 3.0 m and most of the area below 3.9 m. The main high-error zone was limited to the upper-left and middle-left regions. By comparison, Schemes I and III produced larger mean errors (3.3 m and 3.6 m) and more extended high-error areas near the upper-left and upper-central boundaries.

For the third source layer, Scheme II yielded the smallest mean error (2.8 m), keeping most of the monitoring area below 5.3 m, whereas Schemes I and III reached 3.8 m and 3.6 m with high-error concentrations along the lower-left and upper-left boundaries. Overall, under the 0–2% arrival-time-error condition, Scheme II provided the most favorable localization performance across the three source layers.

**Figure 6:**
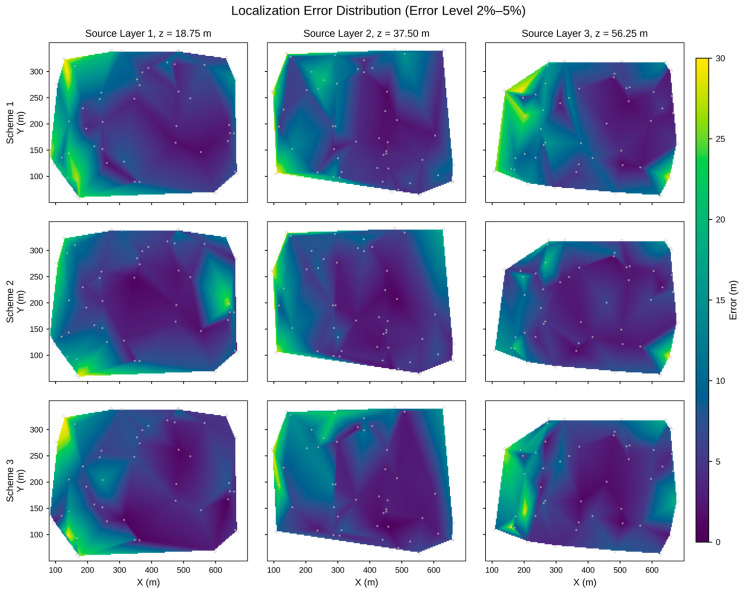
Localization error distribution of different layout schemes under arrival time errors of 2–5%.

When the arrival-time error ranged from 2% to 5%, the localization error distributions of the three monitoring schemes are shown in Figure 6. For the first source layer, Scheme II achieved the lowest mean localization error (8.9 m), with about 85% of the monitoring area remaining below 15.3 m and the larger errors confined mainly to the lower-left and upper-left boundaries. Schemes I and III showed higher mean errors (10.4 m and 9.2 m, respectively), with more pronounced high-error regions along the lower-left and upper-left boundaries.

For the second source layer, Scheme II again provided the most favorable performance, with a mean error of 7.2 m and most of the area below 8.9 m. The main high-error zone was limited to the upper-left and middle-left regions. By comparison, Schemes I and III produced larger mean errors (9.0 m and 7.8 m) and more extended high-error areas near the upper-left and lower-left boundaries.

For the third source layer, Scheme II yielded the smallest mean error (7.6 m), keeping most of the monitoring area below 13.1 m, whereas Schemes I and III reached 10.0 m and 8.5 m with high-error concentrations along the upper-left and lower-left boundaries. Overall, under the 2–5% arrival-time-error condition, Scheme II provided the most favorable localization performance across the three source layers.

**Figure 7:**
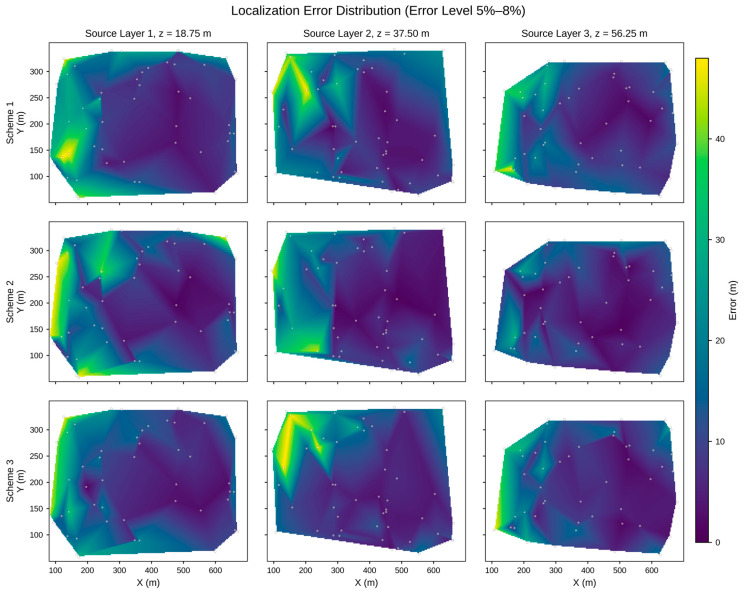
Localization error distribution of different layout schemes under arrival time errors of 5–8%.

When the arrival-time error ranged from 5% to 8%, the localization error distributions of the three monitoring schemes are shown in Figure 7. For the first source layer, Scheme III achieved the lowest mean localization error (15.1 m), with about 85% of the monitoring area remaining below 24.2 m and the larger errors confined mainly to the lower-left and upper-left boundaries. Schemes I and II showed higher mean errors (19.7 m and 17.0 m, respectively), with more pronounced high-error regions along the lower-left and upper-left boundaries.

For the second source layer, Scheme II again provided the most favorable performance, with a mean error of 11.8 m and most of the area below 22.4 m. The main high-error zone was limited to the upper-left and middle-left regions. By comparison, Schemes I and III produced larger mean errors (13.2 m and 13.6 m) and more extended high-error areas near the upper-left and lower-left boundaries.

For the third source layer, Scheme II yielded the smallest mean error (9.7 m), keeping most of the monitoring area below 16.1 m, whereas Schemes I and III reached 14.5 m and 12.7 m with high-error concentrations along the upper-left and lower-left boundaries. Overall, under the 5–8% arrival-time-error condition, Scheme II provided the most favorable localization performance across the three source layers.

**Figure 8:**
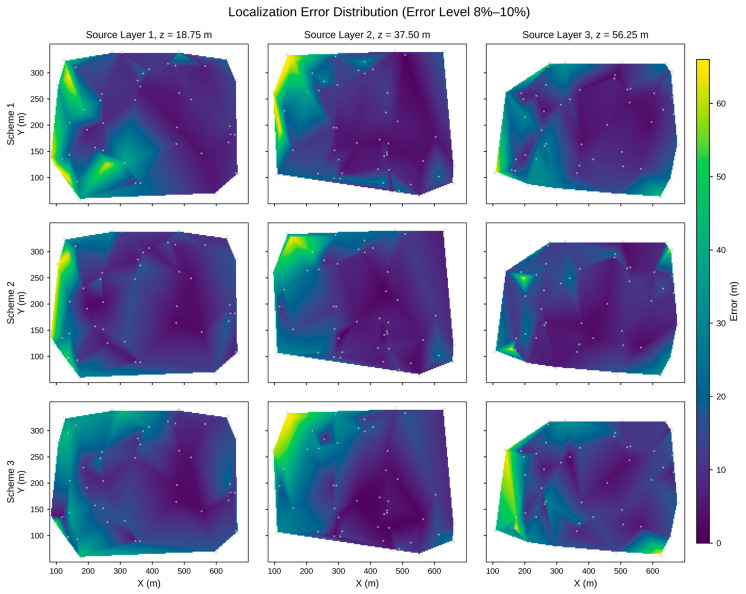
Localization error distribution of different layout schemes under arrival time errors of 8–10%.

When the arrival-time error ranged from 8% to 10%, the localization error distributions of the three monitoring schemes are shown in Figure 8. For the first source layer, Scheme III achieved the lowest mean localization error (18.9 m), with about 85% of the monitoring area remaining below 33.9 m and the larger errors confined mainly to the upper-left and lower-left boundary. Schemes I and II showed higher mean errors (24.1 m and 22.2 m, respectively), with more pronounced high-error regions along the lower-left and upper-left boundaries.

For the second source layer, Scheme II again provided the most favorable performance, with a mean error of 14.4 m and most of the area below 24.1 m. The main high-error zone was limited to the upper-left and lower-left regions. By comparison, Schemes I and III produced larger mean errors (18.8 m and 17.9 m) and more extended high-error areas near the upper-left and lower-central boundaries.

For the third source layer, Scheme II yielded the smallest mean error (16.4 m), keeping most of the monitoring area below 26.7 m, whereas Schemes I and III reached 16.6 m and 19.4 m with high-error concentrations along the lower-left and upper-central boundaries. Overall, under the 8–10% arrival-time-error condition, Scheme II provided the most favorable localization performance across the three source layers.

In addition to the spatial error maps, event-wise robustness metrics were calculated for each layout, including the mean, median, 95th percentile, worst-10% mean localization error, and the proportions of events with localization errors smaller than 10 m and 20 m. To evaluate cross-layer stability, a layer-balance index was defined as the coefficient of variation in the mean localization errors of the three source layers under the same arrival-time error level.

### 3.2. Event-Wise Robustness Statistics

To complement the spatial error maps, event-wise robustness statistics were further calculated for the three layouts under different arrival-time error levels, as summarized in Table 1. The metrics include the mean, median, 95th percentile, worst-10% mean localization error, and the proportions of events with localization errors below 10 m and 20 m.

The statistical results show that Scheme II provides the most favorable overall localization robustness under non-zero arrival-time uncertainty. Compared with Schemes I and III, Scheme II generally yields lower mean localization errors, more stable threshold-based performance, and smaller tail-risk amplification. These results indicate that Scheme II achieves the best balance between localization accuracy and noise resistance from a global event-wise perspective. Table 1 describes the event-wise robustness of the layouts, whereas Table 2 explains these differences from the perspective of geometric configuration.

### 3.3. Geometry-Based Interpretation of Layout Performance

**Figure 9:**
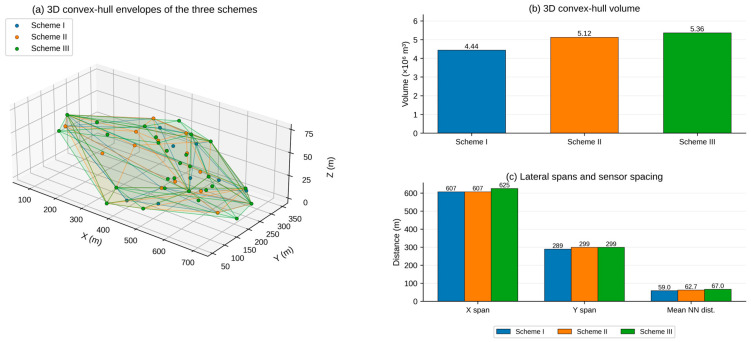
Geometric comparison of the three sensor network schemes: (**a**) overlay of the three-dimensional convex-hull envelopes formed by the 28 sensors of each scheme; (**b**) three-dimensional convex-hull volume; and (**c**) lateral spans (X, Y) and mean nearest-neighbor distance. Scheme III exhibits the largest hull volume and lateral spans, confirming its stronger spatial enclosure, whereas Scheme I is the most compact.

To further interpret the differences in localization performance, several geometry-related metrics of the three layouts were calculated, including the three-dimensional hull volume, spatial spans, and nearest-neighbor distance statistics, as listed in Table 2 and visualized in Figure 9.

The geometry metrics in Table 2 show that Scheme III has the largest three-dimensional spatial enclosure, whereas Scheme II presents a more balanced geometric configuration with moderate enclosure and more favorable node spacing characteristics. Scheme I, by comparison, is relatively compact and exhibits the smallest enclosure capacity among the three layouts. These geometric differences help explain why Scheme III performs better in terms of spatial coverage, while Scheme II yields stronger overall event-wise robustness.

## 4. Discussion

The localization error distributions presented in Section 3 show that the controlling factors of layout robustness vary with the level of arrival-time uncertainty. When the arrival-time error remains within the low-to-moderate range (0–5%), localization performance is mainly influenced by local geometric conditions, including source-to-sensor distance and the intersection geometry of travel paths in specific subregions [13,14]. Under these conditions, layouts with favorable local coverage can achieve lower localization errors.

As the arrival-time error increases to 5–10%, the influence of local geometric advantages becomes less pronounced, and the overall spatial enclosure of the monitoring network plays a more important role in error control [14,15,27]. Layouts with broader three-dimensional coverage are better able to limit the expansion of high-error regions and to maintain more stable localization performance across the monitoring volume. In this stage, the spatial envelope of the sensor network becomes a key factor governing robustness.

This variation in controlling factors also affects layout selection under different source-layer and uncertainty conditions. As summarized in Table 3, the preferred layout is not identical for all source layers or all arrival-time error levels. The results indicate that layout performance is jointly controlled by local geometric matching and global spatial enclosure [17], and that the most suitable configuration depends on the corresponding monitoring horizon and uncertainty level.

It should be noted that, because the deep mining zone of the Woxi mine lies several hundred meters below the surface, sensors can only be installed within existing tunnels, stopes, and accessible openings, and an enclosing surface array is not feasible. The persistently larger errors observed at the periphery of the monitoring area (Figure 5, Figure 6, Figure 7 and Figure 8) are a direct consequence of this constraint: peripheral sources lie near or beyond the edge of the array convex hull, where ray-path intersection angles are unfavorable. Where local development openings permit, spacing sensors laterally outward from the main tunnels or adding nodes near the monitored boundaries is expected to reduce these peripheral errors and to make the error distribution more uniform and is recommended for subsequent in-mine deployment. Finally, a limitation of this study is that the evaluation is based on synthetic events and a homogeneous-velocity assumption; the absolute error values should therefore be interpreted as relative indicators for comparing layouts rather than as field-accuracy predictions, and field microseismic and blasting data will be used in subsequent work to validate and calibrate the selected layout.

## 5. Conclusions

A numerical framework based on localization error analysis was established to compare three candidate sensor network layouts for microseismic monitoring in the Woxi Gold-Antimony-Tungsten Mine under different arrival-time uncertainty levels. The main conclusions are as follows.

(1) Sensor layout geometry strongly influences both the magnitude and the spatial distribution of localization errors. The layout with the lowest global event-wise localization error is not necessarily the one with the strongest three-dimensional spatial enclosure.

(2) The preferred layout varies with the evaluation objective and the arrival-time uncertainty level. From the perspective of global event-wise robustness, Scheme II shows the most favorable overall performance under non-zero arrival-time error conditions. From the perspective of spatial enclosure and inter-layer balance, Scheme III exhibits stronger geometric coverage, especially under higher uncertainty levels.

(3) As arrival-time uncertainty increases, the controlling factors of localization performance shift from local geometric advantages to the overall spatial enclosure of the monitoring network. Layouts with broader three-dimensional coverage are more effective in limiting the expansion of high-error regions and maintaining stable localization performance under high-noise conditions.

These results provide a geometry-based reference for the preliminary design and subsequent refinement of a mine-scale microseismic monitoring system in the Woxi mine. For practical deployment, Scheme II is recommended as the baseline configuration under the expected low-to-moderate arrival-time uncertainty, as it provides the lowest mean localization error and the most stable threshold-based performance; when higher timing uncertainty or stronger vertical coverage is required, Scheme III is preferable because of its larger three-dimensional enclosure and best inter-layer balance. It should be noted that the present evaluation is based on synthetic events and a homogeneous-velocity assumption, so the absolute error values should be interpreted as relative indicators for comparing layouts rather than as field-accuracy predictions. In the next phase, the recommended layout will be installed in the deep section, and the synthetic predictions will be validated against field microseismic and controlled-blasting records, with supplementary monitoring nodes added in the peripheral high-error zones identified in this study.

## Figures and Tables

**Table 1:** Event-wise robustness metrics of the three layouts.

Scheme	Arrival-Time Error	Mean (m)	Median (m)	P95 (m)	Worst 10% Mean (m)	<10 m (%)	<20 m (%)
Scheme I	0–2%	3.68	2.67	9.49	11.15	96.67	99.33
Scheme II	0–2%	3.33	2.93	7.70	9.02	96.67	100.00
Scheme III	0–2%	3.88	2.42	10.71	12.89	94.00	99.33
Scheme I	2–5%	10.31	7.84	26.53	29.64	63.33	86.00
Scheme II	2–5%	8.04	5.68	24.00	26.80	76.00	91.33
Scheme III	2–5%	8.82	5.54	24.65	28.60	70.00	91.33
Scheme I	5–8%	14.51	9.62	36.83	48.41	52.00	79.33
Scheme II	5–8%	12.60	8.49	41.66	44.89	57.33	86.00
Scheme III	5–8%	14.07	11.01	37.38	43.70	46.67	82.00
Scheme I	8–10%	18.74	11.46	58.95	70.67	41.33	72.67
Scheme II	8–10%	16.88	11.13	49.06	55.14	45.33	70.67
Scheme III	8–10%	18.69	12.40	54.35	61.38	40.67	69.33

**Table 2:** Geometry metrics of the three layouts.

Scheme	X Span (m)	Y Span (m)	Z Span (m)	3D Hull Volume	Mean Nearest-Neighbor Distance (m)	Std. of Nearest-Neighbor Distance (m)
Scheme I	607.32	289.15	75	4.44 × 10^6^	58.96	19.92
Scheme II	607.32	299.17	75	5.12 × 10^6^	62.74	23.02
Scheme III	625.34	299.17	75	5.36 × 10^6^	66.97	21.72

**Table 3:** The optimal sensor monitoring network selection for each layer of simulated microseismic under different arrival time error levels.

Error Range	Source Layer 1	Source Layer 2	Source Layer 3
0%	Scheme I	Scheme II	Scheme III
0~2%	Scheme III	Scheme I	Scheme III
2~5%	Scheme III	Scheme II	Scheme III
5~8%	Scheme II	Scheme III	Scheme I
8~10%	Scheme III	Scheme III	Scheme III

## Data Availability

The original contributions presented in this study are included in the article. Further inquiries can be directed to the corresponding author.
